# Partial analytical validation of the VetScan cPL rapid test

**DOI:** 10.1111/vcp.12796

**Published:** 2019-11-06

**Authors:** Joerg M. Steiner, Phillip Guadiano, Robynne R. Gomez, Jan S. Suchodolski, Jonathan A. Lidbury

**Affiliations:** ^1^ Gastrointestinal Laboratory Texas A&M University College Station TX USA

**Keywords:** canine pancreatic lipase immunoreactivity, immunoassay, pancreatitis, point‐of‐care

## Abstract

**Background:**

Serum canine pancreatic lipase immunoreactivity (cPLI) concentrations have become the standard laboratory test used to diagnose canine pancreatitis. Recently, a new point‐of‐care assay for cPLI, the VetScan cPL rapid test (VetScan cPL), has become available, but analytical validation data have not yet been published.

**Objective:**

This study aimed to perform a partial analytical validation of the VetScan cPL.

**Methods:**

Leftover serum samples from a diagnostic laboratory were used. Adherence to the manufacturer's guidelines, linearity, repeatability, and reproducibility were evaluated. Results of the VetScan cPL were correlated with the Spec cPL results.

**Results:**

Observed‐to‐expected ratios for dilutional parallelism ranged from 77.4% to 162.9% (mean 119.3%). Intra‐assay and inter‐assay variabilities ranged from 16.9% to 36.7% (mean 25.1%) and from 14.1% to 51.2% (mean 31.8%), respectively. Adherence to the manufacturer's specification regarding results within ± 60 µg/L of the Spec cPL result was only achieved for 39% of the measurements. The VetScan cPL and Spec cPL correlation showed a Spearman's *r* of .758 for 29 data pairs.

**Conclusions:**

Under the conditions of this study, the VetScan cPL did not adhere to the manufacturer's specifications for most measurements. Also, the VetScan cPL showed suboptimal linearity and was not precise. In conclusion, the VetScan cPL failed basic analytical validation.

## 1 INTRODUCTION

Pancreatitis is common in dogs.^1^ These dogs can present with a wide range of clinical presentations from subclinical disease to mild chronic disease to severe acute disease. This range of clinical presentations makes the diagnosis of pancreatitis challenging. Currently, abdominal ultrasonography and the measurement of serum pancreatic lipase concentrations are considered to be the most useful diagnostic modalities for canine pancreatitis.^2^ A variety of ultrasonographic changes have been reported in dogs with pancreatitis.^3^, ^4^ However, these findings can be subjective, and the overall diagnostic efficacy of abdominal ultrasonography for dogs with pancreatitis is dependent on the disease severity, equipment quality, operator experience, and level of suspicion for pancreatitis by the operator.

In contrast to assays for lipase activity, serum pancreatic lipase immunoreactivity (cPLI) measurements are highly specific for the detection of pancreatic lipase.^5^ In one study that evaluated serum cPLI concentrations in shelter dogs that had been euthanized for other reasons, the specificity of the serum cPLI concentration was 95.7%.^5^ Measuring cPLI concentrations is also highly sensitive for the diagnosis of pancreatitis, though reported sensitivities have depended on the clinical presentation and study design.^6^, ^7^, ^8^ Until recently, the only commercially available assays measuring cPLI were the Spec cPL and SNAP cPL (IDEXX Laboratories). The Spec cPL is a laboratory‐based ELISA, using a recombinant antigen (recombinant canine pancreatic lipase) and a monoclonal antibody directed against native canine pancreatic lipase.^9^ The analytical validation of this assay has been reported in the peer‐reviewed literature.^9^ The Spec cPL assay was reported to be linear upon dilution with a working range of 30‐2000 µg/L.^9^ Intra‐assay variability for three samples and 12 repeated measurements were reported to be 7.8%, 9.0%, and 11.2%, and the inter‐assay variability for three samples and five repeat measurements were reported to be 3.8%, 7.6%, and 5.6%.^9^ The SNAP cPL is a point‐of‐care semi‐quantitative device that is easy to perform and results in either a "normal" read (ie, the associated Spec cPL is in the reference interval [RI] of <200 µg/L) or an "abnormal" read (ie, the associated Spec cPL is either suggestive of pancreatitis or in the questionable range).^6^, ^10^ Abaxis has recently released a point‐of‐care test for cPLI that is also based on a rapid assay device that is being read by a reader connected to a smartphone. To the authors' knowledge, no analytical validation data have been provided for this new assay platform either in the peer‐reviewed literature or the assay documentation. Thus, the goal of the current study was to perform a partial analytical validation of this new assay platform. This study is especially timely as a recent report suggested that the VetScan cPL correlated well with the Spec cPL and the diagnostic bin of the Spec cPL.^11^ However, in that study, the assay was not performed as it would be used in clinical practice, but serum samples were sent to a central research laboratory where all samples were analyzed.^11^ Because the comparison of the VetScan cPL with the Spec cPL has been reported previously, this study did not perform a method comparison study. The Spec cPL results are reported as reference points for the VetScan cPL results.

## 2 MATERIALS AND METHODS

### 2.1 Samples

All samples used for the partial validation of the VetScan cPL assay were from leftover serum samples that had been submitted to the Gastrointestinal Laboratory at Texas A&M University. All samples had originally been analyzed at the GI Lab, and then the leftover serum samples were frozen at −20°C for up to 4 months. However, all Spec cPL measurements referred to in this manuscript were performed at the same time as the VetScan cPL measurements. Before analyses, all samples were thawed and brought to room temperature, as suggested by the manufacturer.

### 2.2 The Vue analyzer and VetScan cPL assay

All VetScan cPL measurements were taken using four different Vue Analyzers obtained from Abaxis (Union City, California). The Vue is a point‐of‐care analyzer that reads proprietary lateral flow devices, such as the VetScan cPL device. The assay is based on lateral flow technology that uses affinity‐purified antibodies directed against canine pancreatic lipase, which is bound to colloidal gold particles. Serum samples are applied to the device, and the pancreatic lipase in the sample binds to antibody‐coated gold particles. These complexes are then captured by a secondary antibody on the test strip. The accumulation of the captured gold particle/enzyme complex causes the color indicator to become visible on the test line. The signal is further amplified by the use of a competitive antibody calibration scheme employed on the control line. The darkness of the lines is quantified by densitometric analysis in the VetScan VUE (information taken from the product insert of the VetScan cPL). The working range of the assay is 50‐60 µg/L to <700 to <800 µg/L. The assay marketing material suggests that the assay is quantitative and leads to results that are within a band of ±60 µg/L of the Spec cPL result. However, while most readers would display and report results as ±60 µg/L, some readers report results as ±50 µg/L.

### 2.3 Linearity

The linearity of the assay was assessed by measuring dilutional parallelism of six canine serum samples of high‐quality undiluted and at dilutions of 1:2, 1:4, and 1:8 with a pooled nonlipemic serum sample with an undetectable serum Spec cPL concentration.

### 2.4 Effect of lipemia

For this experiment, we evaluated 3 naturally hypertriglyceridemic serum samples with serum triglyceride concentrations of 525, 580, and 1319 mg/dL undiluted, and at dilutions of 1:2 and 1:4.

### 2.5 Reproducibility

Intra‐assay variability was tested with three high‐quality serum samples, designated Samples 1, 2, and 3, evaluated 10 times on four different VUE analyzers. Measurements were performed on the same analyzer during a single session on a single day. For this study, an intra‐assay variability of ≤10% was considered acceptable, an intra‐assay variability 10% < %CV ≤ 20% was considered poor but acceptable, and an intra‐assay variability of >20% was considered unacceptable.

### 2.6 Repeatability

For the assessment of inter‐assay variability, 10 high‐quality serum samples were measured eight times on the same analyzer on different days (measurements were performed on consecutive days with no measurements taken on weekend days). Serum sample concentrations spanned the lower third of the Spec cPL assay working range since our previous findings showed that serum samples with a serum Spec cPL concentration >700‐800 µg/L would often read outside the VetScan cPL assay working range. The 10 samples had Spec cPL concentrations of 196, 227, 254, 322, 360, 388, 400, 477, 566, and 594 µg/L. Four different analyzers were used for this experiment, and eight serum samples were analyzed eight times on a single analyzer on eight different days, while two serum samples were analyzed eight times on eight different days on each of the four analyzers. Leftover serum sample concentrations were chosen throughout the working range of the VetScan cPL assay. They were then aliquoted, and each aliquot was frozen in a separate sample tube at −20°C. Samples were removed from the freezer and thawed immediately prior to analysis. For this study, an inter‐assay variability of ≤10% was considered acceptable, an inter‐assay variability 10% ≤ %CV ≤ 20% was considered poor but acceptable, and an inter‐assay variability of > 20% was considered unacceptable.

### 2.7 Adherence to the manufacturer's specifications

To determine whether the new VetScan cPL adhered to the manufacturer's specification that the results would be within ±60 µg/L of the Spec cPL assay, each nonlipemic measurement was assessed. It should be noted that one VUE analyzer read the result as ±50 µg/L of the Spec cPL assay, but the more conservative criterion of ±60 µg/L was used for these assessments. Spec cPL concentrations were determined and used as the target values. However, only VetScan Vue measurements were used to evaluate this criterion and were measured close to the same time as that of the Spec cPL concentrations. Thus, for the inter‐assay variability determinations where samples were run multiple times on consecutive days, only the initial Spec cPL concentration was used.

### 2.8 Correlations

While we did not run a correlation study per se, our experiments created a set of data pairs that were then used to assess the correlation. The values for undiluted samples from the dilutional parallelism and lipemia experiments were used. Also, the first data point, each from the intra‐ and inter‐assay variability experiments was used for this analysis, which generated a total of 34 data pairs. After excluding data pairs with results that were outside the working range for either assay (five data pairs had results that were outside the working range of the Vet Scan assay, one data pair from the intra‐assay variability study had a VetScan cPL of <60 µg/L and a Spec cPL of 120 µg/L, one data pair from the inter‐assay variability study had a VetScan cPL of <50 µg/L and a Spec cPL of 254 µg/L, one data pair from the inter‐assay variability study had a VetScan cPL of <60 µg/L and a Spec cPL of 196 µg/L, and two data pairs from the dilutional parallelism study had a VetScan cPL of >800 µg/L and a Spec cPL of 1265 and 1287 µg/L, respectively), a total of 29 data pairs were selected for analysis.

### 2.9 The statistical methods

For statistical analyses, single results outside the assay working range (eg, <60 µg/L) were transcribed as one unit above or below the working range limit (eg, 59 µg/L). However, sample sets that had results mostly outside of the working range (eg, all 10 samples <60 µg/L) were not used for the statistical analyses. Repeated measure ANOVA was used to assess differences among the different analyzers.

All statistical comparisons were performed using a statistical software package (GraphPad Prism 6.07). The level of statistical significance was set at .05 for all statistical comparisons. Spearman's correlation coefficient (r) was calculated using a statistical software package (GraphPad Prism).

## 3 RESULTS

### 3.1 Linearity

The average observed‐to‐expected (O/E) ratio for a total of 13 observations was 119.3% (±SD: 28.7%). Of those O/E ratios, two were between 90% and 110% (ideal range), four were between 80% and 120% (acceptable range), and seven were outside the acceptable range (77.4, 123.4, 130.2, 140.0, 150.6, 159.1, and 162.9%) (Table 1).

**Table 1:** This table shows the dilutional parallelism for six canine serum samples. The first sample was outside the working range of the analyzer for the neat sample, and the sample diluted 1:2. Therefore, the sample diluted 1:4 was used as the baseline reference for the calculation of observed/expected ratios (O/E ratios). The 1:4 and 1:8 dilutions of Sample 6 were also outside the working range of the analyzer

Sample#	Dilution	Spec cPL	Observed	Expected	O/E ratio
		µg/L	µg/L	µg/L	%
1	Neat	1287	>800		
	1:2		>800	N/A	N/A
	1:4		617	N/A	N/A
	1:8		328	309	106.3
2	Neat	1265	>800		
	1:2		757		N/A
	1:4		320	379	84.5
	1:8		204	189	107.8
3	Neat	621	496		
	1:2		323	248	130.2
	1:4		96	124	77.4
	1:8		101	62	162.9
4	Neat	548	548		
	1:2		318	274	116.1
	1:4		169	137	123.4
	1:8		109	69	159.1
5	Neat	367	542		
	1:2		218	271	80.4
	1:4		152	136	112.2
	1:8		102	68	150.6
6	Neat	182	110		
	1:2		77	55	140.0
	1:4		<50	28	N/A
	1:8		<50	14	N/A
Mean					119.3
SD					28.7

### 3.2 The effects of lipemia

For this experiment, we evaluated serum samples from three hypertriglyceridemic dogs with serum triglyceride concentrations of 525, 580, and 1319 mg/dL. For two samples, the VetScan cPL rapid test neat measurement was outside the analyzer working range of 700 µg/L, while the third sample had a concentration of 320 µg/L. Concentrations for the 1:2 and 1:4 dilutions were 292 µg/L with an O/E ratio of 182.5% and 65 µg/L with an O/E ratio of 81.3%, respectively. Both measurements were outside the preferred range of ±10%, and one was outside the acceptable range of ±20%. For the other two samples that had undiluted cPLI concentrations above the upper limit of the working range of the VetScan cPL assay, the concentrations for the 1:2 dilution were 334 µg/L for both samples. For the 1:4 dilution, the concentrations were 107 µg/L (O/E ratio, 64.1%) and 449 µg/L (O/E ratio, 268.9%), respectively, and both concentrations fell into the unacceptable range (Table 2).

**Table 2:** Dilutional parallelism of lipemic serum samples from three hyperlipidemic dogs. Two samples had VetScan cPL results outside the assay working range, so the 1:2 dilution was used as a baseline reference. All observed/expected ratios (O/E ratios) were outside the range of 80%‐120%, which is generally considered to be acceptable

Sample#	Dilution	Triglyceride	Spec cPL	VetScan cPL	O/E ratio
		mg/dL	µg/L	µg/L	%
1	Neat	525	337	320	
	1:2			292	**182.5**
	1:4			65	**81.3**
2	Neat	580	737	>700	
	1:2			334	N/A
	1:4			107	**64.1**
3	Neat	1319	1368	>700	
	1:2			334	N/A
	1:4			449	**268.9**
Mean					149.2
SD					95.4

### 3.3 Repeatability

The serum Spec cPL concentrations for the repeatability study had one sample each with Spec cPL concentrations of 120 µg/L (within the RI; Sample 1), 233 µg/L (in the questionable range; Sample 2), and 588 µg/L (in the suggestive for pancreatitis range; Sample 3). For Sample 1, the %CVs were 19.4, 22.9, 23.0, and 31.5%, for the four different analyzers, which represented an unacceptable %CV for three out of the four analyzers (%CV > 20%, unacceptable). One out of four analyzers had %CVs that were acceptable but poor (10% > %CV ≤ 20%). Sample 1 had values that ranged from 54 to 188 µg/L with an overall mean of 106 µg/L. Also, 33 Sample 1 measurements (82.5%) met the manufacturer's specifications of being in the range of Spec cPL ± 60 µg/L while seven did not, and all results fell into the RI. The four analyzers produced significantly different results from one another with sample means of 66, 105, 122, and 133 µg/L; *P* = .0037 (Table 3).

**Table 3:** Repeatability (intra‐assay variability) of three serum samples from dogs analyzed on four different analyzers performed 10 times in one run. None of the variabilities was less than 10%, which is a general target value for repeatability, and only three of the 12 variabilities were within the "poor but acceptable" range of 10%‐20%

**Sample#**	**Spec cPL**	**Analyzer**	**Measurements**	**%CV**
	**µg/L**			
1	120	1	10	22.9
		2	10	19.4
		3	10	31.5
		4	10	23.0
2	233	1	10	26.0
		2	10	34.7
		3	10	30.3
		4	10	22.3
3	588	1	10	18.7
		2	10	34.6
		3	10	21.0
		4	10	16.9
Mean				25.1
SD				6.2

Abbreviations: SD, standard deviation; %CV, percent coefficient of variation.

Sample 2 had %CVs of 22.3, 26.0, 30.3, and 34.7% for the four different analyzers, which would be considered unacceptable for all four analyzers. For this sample, the values ranged from 56 to 423 µg/L, with an overall mean of 232 µg/L. Twenty‐one measurements (52.5%) met the manufacturer's specifications of being in the range of the Spec cPL ± 60 µg/L, while 19 (47.5%) did not. For Sample 2, with a Spec cPL in the questionable range, the VetScan cPL rapid test results had 25 measurements within the RI, 14 measurements in the questionable range, and one measurement in the range suggestive for pancreatitis. The four analyzers produced significantly different results from one another with sample means of 142, 248, 255, and 284 µg/L, *P* = .0005.

Sample 3 had %CVs of 16.9, 18.7, 21.0, and 34.6% for the four different analyzers, which would be considered unacceptable for two analyzers and acceptable but poor for the other two. For this sample, values ranged from 175 to >700 µg/L, with an overall mean of 536 µg/L. Only 11 of the measurements (27.5%) met the manufacturer's specifications of being in the range of the Spec cPL ±60 µg/L, while 29 (72.5%) did not. For this sample, with a Spec cPL in the range of being suggestive for pancreatitis, the VetScan cPL rapid test results had one measurement within the RI, three measurements in the questionable range, and 36 measurements in the range suggestive for pancreatitis. The four analyzers produced significantly different results from one another with sample means of 441, 557, 568, and 577 µg/L; *P* = .014.

### 3.4 Reproducibility

**Figure 1:**
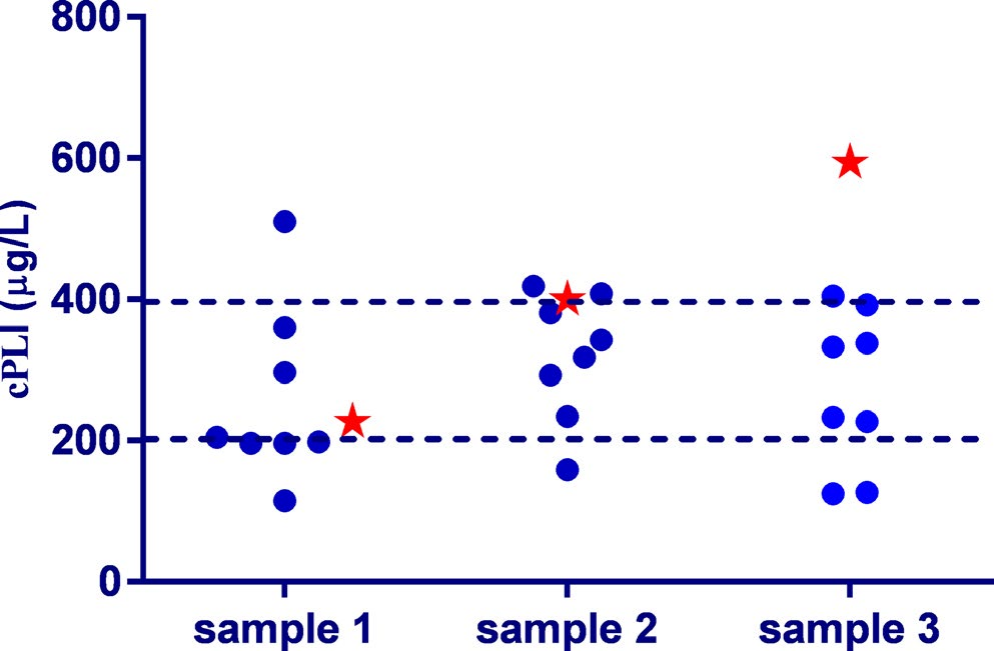
Individual reproducibilities for three canine serum samples are shown in this graph. Samples 1, 2, and 3 had a Spec cPL of 227 µg/L with a mean VetScan cPL of 260 µg/L and a %CV of 48.3%, a Spec cPL of 400 µg/L with a mean VetScan cPL of 319 µg/L and a %CV of 28.0%, and a Spec cPL of 594 µg/L with a mean VetScan cPL of 273 µg/L and a %CV of 40.8%, respectively. Blue dots depict the VetScan measurements, while red stars represent the Spec cPL measurements. The broken lines reflect the cutoff values among the three diagnostic bins of 200 µg/L and 400 µg/L

For inter‐assay variability, one of 10 samples (Spec cPL = 196 µg/L) read <60 µg/L for every measurement. The %CV for this sample could not be assessed (Table 4; Figure 1). For another sample (Spec cPL = 254 µg/L), five measurements were <50 µg/L, and only three other measurements resulted in values within the working range of the assay. Many of the samples spanned all three diagnostic bins of the assay. For example, one sample, with a Spec cPL of 566 µg/L, had two results within the RI (109 and 152 µg/L), four in the questionable range (247, 270, 297, and 392 µg/L), and two in the range suggestive for pancreatitis (467 and 477 µg/L). Only four of the samples had measurements in a single diagnostic bin, seven samples spanned two diagnostic bins, and five samples spanned all three diagnostic bins. To illustrate these findings further, the actual measurements for the three samples are provided in Figure 1. Overall, inter‐assay variability was high, ranging from 14.1% to 51.2% (mean %CV for all samples 31.8%; with a total of 14 data points).

**Table 4:** Reproducibility (inter‐assay variability) of 10 samples analyzed on four different analyzers (two samples were analyzed on all four analyzers, and eight samples were analyzed on one analyzer). None of the %CVs were less than 10%, which is a general target value for reproducibility. Only three of the 14 variabilities were within the "poor but acceptable" range of 10%‐20%. The samples that are shown in red are also depicted in Figure 1

**Sample#**	**Spec cPL**	**Analyzer**	**Measurements**	**%CV**
	**µg/L**			
1	227	1	8	48.3
		2	8	35.1
		3	8	37.1
		4	8	37.8
2	388	1	8	20.4
		2	8	22.1
		3	8	19.4
		4	8	51.2
3	196	1	8	N/A
4	254	2	8	N/A
5	322	1	8	27.0
6	360	3	8	18.9
7	400	4	8	28.0
8	477	1	8	14.1
9	566	2	8	45.2
10	594	2	8	40.8
Mean				31.8
SD				12.0

Abbreviations: SD, standard deviation; %CV, percent coefficient of variation.

### 3.5 Adherence to the manufacturer's specifications

A total of 252 measurements were assessed for adherence to manufacturer's specifications, which would require the results of the VetScan cPL to be within a range of the Spec cPL result ±60 µg/L. Adherence to this specification was only achieved for 99 measurements (39%) but was not achieved for the majority of the measurements (153 samples or 61%).

### 3.6 Correlations 

**Figure 2:**
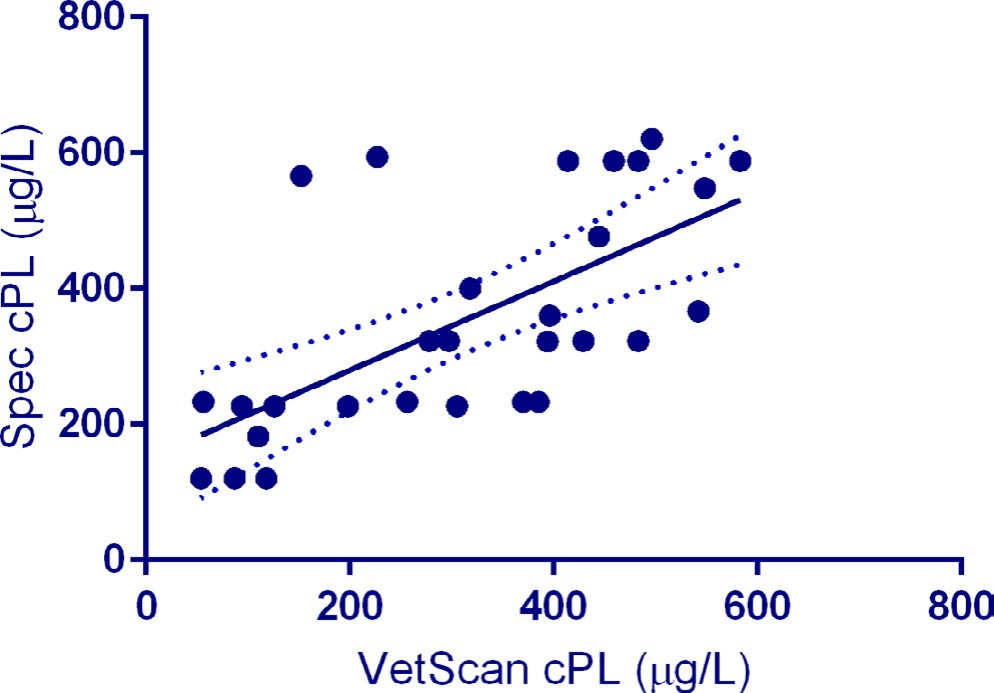
The correlation of the VetScan cPL rapid test with the Spec cPL shows a relatively large variability (see regression line with 95% confidence interval) between results of the two assays measuring the same serum pancreatic lipase analyte, while the Spearman r showed a statistically significant correlation

**Figure 3:**
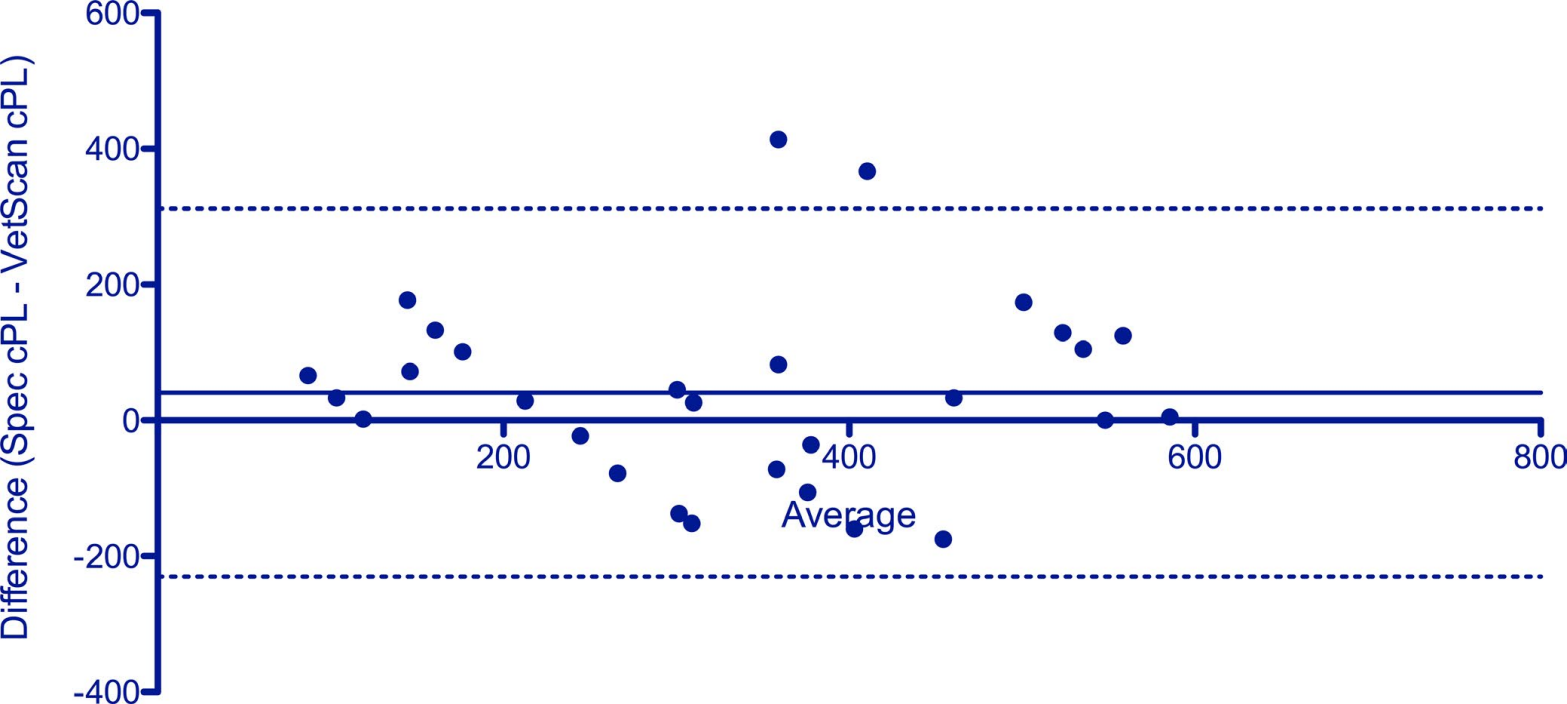
A Bland‐Altman plot shows a mean bias of 40.7 µg/L (solid line) and 95% limits of agreement from −239.4 to 311.7 µg/L (dashed lines). Most variation between the results of the two different assays was random as demonstrated by the wide limits of agreement; and therefore, correction with a constant or proportional adjustment factor would not improve performance

Our studies created several data pairs that could be used for rudimentary correlation assessments. The Spearman *r* for 29 data sets was 0.722 (*P* < .0001) (Figure 2). The Bland‐Altman plot (Figure 3) showed a bias of 40.7 ± 138.3 µg/L (95% limits of agreement: −230.4 to 311.7 µg/L). No correlation between the difference (Spec cPL – VUE) and average of the two measurements (*P* = .9428) was found on the linear regression analysis, which suggested that proportional bias did not exist among the assay results.

## 4 DISCUSSION

In this study, we found that the Abaxis VetScan cPL assay, for measurement of serum cPLI concentrations, showed poor linearity, repeatability, and reproducibility when tested on a single occasion as is usually performed by veterinarians in general practice.

A variety of protocols for analytical validation of newly developed assays exist. Many assays, such as radioimmunoassays and enzyme‐linked immunosorbent assays (ELISAs), intrinsically use a duplicate or even triplicate approach to evaluate standards and assess unknown samples. The VetScan cPL assay evaluated in this study only uses a single measurement to determine each sample result. Thus, to mirror conditions, where a veterinarian would assess each sample only once with this assay, we chose to only analyze each sample once in this study. Analyzing samples in duplicate or triplicate fashion would not have provided meaningful results for the analytical performance of the assay in relation to general veterinary practice.

As mentioned previously, the aim of this study was not to compare the diagnostic specificity and/or sensitivity of the VetScan cPL assay to that of the Spec cPL but rather the partial analytical validation of the VetScan cPL using the Spec cPL as a reference point since this assay has been analytically validated.^9^ Of the other three cPLI assays described in the literature, two are no longer available, and the third one failed analytical validation.^9^, ^12^, ^13^, ^14^

The VetScan cPL rapid test showed limited linearity. This was especially significant since the working range of the assay is relatively narrow (50 or 60 µg/L to 700 or 800 µg/L depending on the VUE analyzer used) when compared with the Spec cPL (30‐2,000 µg/L). Therefore, to be able to monitor disease progression using serum cPLI concentrations, dilutions would be required for many samples.^15^ It should be noted that Abaxis does not currently recommend a protocol for the dilution of samples with high results; thus, based on the narrow assay working ranges, a sample dilution protocol should be developed that could improve the linearity of the assay. Evaluation of assay linearity was complicated by the fact that two of the six undiluted serum samples read outside the working range of the assay.

The standard method to test the influence of lipemia on serum biochemical assays is the addition of various amounts of Intralipid to various serum samples.^16^, ^17^ However, previous studies in both humans and dogs have shown that adding Intralipid might have a much greater impact on lipase assays than naturally occurring hypertriglyceridemia.^16^, ^18^ Thus, in a previous study, we attempted to assess the impact of natural hypertriglyceridemia on serum lipase activities by diluting samples from patients with spontaneous hypertriglyceridemia.^16^ In the current study, the linearity of the VetScan cPL rapid test for the hypertriglyceridemic samples was worse than was observed for high‐quality samples, which suggests that while lipemia does not have a predictable statistically significant effect on the VetScan cPL results, it can impact the measurement of canine‐specific lipase using this assay. However, since only a few samples were analyzed in this study, further samples should be assessed to confirm these results. This is important because serum samples from dogs with acute pancreatitis are often lipemic. Thus, the impact of lipemia on assay performance might also affect clinical decisions. Therefore, the VetScan cPL rapid test should be used with caution in lipemic dogs until further studies on the impact of lipemia with this assay have been performed.

We designated acceptable and unacceptable ranges for the inter‐assay variability measurements because no universally accepted performance goal standards for diagnostic assays exist. We chose these goals based on the fact that most commercial assay manufacturers achieve and report intra‐assay variabilities of <10%. Overall, repeatability of the VetScan cPL assay was poor. For the first sample with a Spec cPL of 120 µg/L, values ranged from 54 to 188 µg/L with an overall mean of 106 µg/L. One could argue that this high variability is not clinically important as it did not change the diagnosis since all 40 measurements were within the reference interval. However, for samples that are close to assay cutoff values, this high variability could have a much larger impact. For Sample 2, the intra‐assay results showed that while the overall sample mean was close to the Spec cPL value, the variability was unacceptably high, which showed how the lack of repeatability could have an impact on clinical decisions. Sample 3 results also had highly variable repeatability with a sample mean that was close to the Spec cPL value. Overall, the assay fails basic sample repeatability and analyzer variability (nine of 12 intra‐assay variability experiments showed a %CV of >20%).

The reproducibility of the VetScan cPL rapid assay was also poor often resulting in concentrations that were in different diagnostic bins for the same sample. Since there would be no reason to repeat the measurement for a sample in a clinical setting, a veterinarian could easily misdiagnose a patient as not having pancreatitis or as having pancreatitis based on this poor reproducibility.

Since Abaxis has not published any data on the repeatability or reproducibility of the VetScan cPL rapid assay, it is unclear why repeatability and reproducibility were poor in our study. One possible explanation is that the assay kits are affected by transport and storage conditions. According to the manufacturer, the VetScan cPL rapid test kits do not require refrigeration during storage. While the assay kits were kept at room temperature in our laboratory, it is conceivable that this could have affected repeatability and reproducibility. However, regardless of the reasons, the poor repeatability and reproducibility identified in our study suggest that under normal veterinary practice conditions, the VetScan cPL does not produce precise diagnostic results.

The limited correlation study (*r* = .722) could be considered high for two different clinical pathology variables that assess similar physiologic functions (eg, serum creatinine and BUN concentrations to assess renal function), but would be considered poor for trying to correlate two assays that measure the same analyte. As the calculated bias of 40.66% µg/L between the two assays was apparently random with no proportional bias, it would not be possible to correct the results with a constant or proportional adjustment factor.

One limitation of this study is that all VetScan cPL results were only obtained with single measurements. As indicated in the introduction of this manuscript, validation studies for clinical pathology analytes often use duplicate or even triplicate measurements. However, the aim of this study was to describe analytical validation parameters under veterinary practice conditions, where a veterinarian would only run a patient sample on VetScan cPL once. Further studies using duplicate and/or triplicate measurements could provide further analytical validation data for this assay.

In summary, the Abaxis VetScan cPL assay for measurement of serum cPLI concentrations shows poor linearity, repeatability, and reproducibility when tested on a single occasion as usually performed by veterinarians in general practice. Intra‐ and especially inter‐assay variability were poor, and most sample results were identified in more than one diagnostic bin upon repeat analysis. The manufacturer's specification, suggesting that the VetScan cPL provides results within ±60 µg/L of the Spec cPL result was not confirmed by our studies as we often identified large differences with the serum Spec cPL concentrations. Further validation of this assay is, therefore, needed before this assay's clinical usefulness can be appropriately studied.

## DISCLOSURES

All authors are employed by or affiliated with the Gastrointestinal Laboratory at Texas A&M University, which performs cPLI testing on a fee‐for‐service basis. Dr Steiner also serves as a paid consultant for IDEXX Laboratories, the manufacturer of Spec cPL and SNAP cPL.
